
JOURNAL INFORMATION
==============================
NLM Title Abbreviation: Wirtschaftsdienst
Journal ID: Wirtschaftsdienst
NLM Journal ID: 101086612

Wirtschaftsdienst (Hamburg, Germany : 1949)

ISSN: 0043-6275

ARTICLE INFORMATION
==============================
PMCID: PMC7368595
PMID: 32834166
DOI: 10.1007/s10273-020-2693-4
Article ID: 2693
Article version: 1
Subjects: Zeitgespräch

Ein Investitionspaket ist das beste Konjunkturpaket

Krebs Tom tkrebs@econ.uni-mannheim.de

grid.5601.2 0000 0001 0943 599X Abteilung Volkswirtschaftslehre, Universität Mannheim, L7, 3-5, 68161 Mannheim, Deutschland
Electronic publication date: 2020 Jul 18
Print publication date: 2020
Volume: 100
Issue: 7
First page: 497
Last page: 500
Copyright: © Der/die Autor(en) 2020
License: Open Access Dieser Artikel wird unter der Creative Commons Namensnennung 4.0 International Lizenz veröffentlicht, welche die Nutzung, Vervielfältigung, Bearbeitung, Verbreitung und Wiedergabe in jeglichem Medium und Format erlaubt, sofern Sie den/die ursprünglichen Autor(en) und die Quelle ordnungsgemäß nennen, einen Link zur Creative Commons Lizenz beifügen und angeben, ob Änderungen vorgenommen wurden.
Die in diesem Artikel enthaltenen Bilder und sonstiges Drittmaterial unterliegen ebenfalls der genannten Creative Commons Lizenz, sofern sich aus der Abbildungslegende nichts anderes ergibt. Sofern das betreffende Material nicht unter der genannten Creative Commons Lizenz steht und die betreffende Handlung nicht nach gesetzlichen Vorschriften erlaubt ist, ist für die oben aufgeführten Weiterverwendungen des Materials die Einwilligung des jeweiligen Rechteinhabers einzuholen.
Weitere Details zur Lizenz entnehmen Sie bitte der Lizenzinformation auf http://creativecommons.org/licenses/by/4.0/deed.de.
License URL: https://creativecommons.org/licenses/by/4.0/

issue-copyright-statement: © The Author(s) 2020

==============================

Die deutsche Wirtschaft befindet sich derzeit in der tiefsten Rezession der Nachkriegsgeschichte. Es sind solche Zeiten der allgemeinen Verunsicherung, die staatliches Krisenmanagement und aktive Konjunkturpolitik erforderlich machen. Ohne staatliche Impulse besteht die Gefahr, dass Wirtschaft und Gesellschaft dauerhaften Schaden erleiden – der Hysterese-Effekt einer Rezession. Die Weltwirtschaftskrise Anfang der 1930er Jahre hat eindrucksvoll gezeigt, welche erheblichen gesellschaftlichen Schäden falsche wirtschaftspolitische Entscheidungen verursachen können.

Doch die Krise kann auch eine Chance sein, strukturelle Schwächen zu überwinden und die notwendige sozial-ökologische Transformation voranzutreiben. Denn bereits vor der Krise stand fest, dass Deutschland ökologischer Wirtschaften muss, um die Klimaziele zu erreichen. Darüber hinaus ist in großen Teilen der Gesellschaft die Chancenungleichheit hoch und der Wettbewerb unfair. Eine Konjunkturpolitik, die gleichzeitig Transformationspolitik ist, kann hier wichtige Impulse zur Stärkung der Generationengerechtigkeit und sozialen Gerechtigkeit geben.

Die Bundesregierung muss in der Corona-Krise also auf einen Dreiklang setzen: Stabilisierungspolitik zur Verhinderung des wirtschaftlichen Absturzes, Konjunkturpolitik zur Stärkung der wirtschaftlichen Erholung und Transformationspolitik für eine bessere Zukunft. Der vorliegende Beitrag beschreibt die Grundzüge einer Wirtschaftspolitik, die diesen Dreiklang erreichen kann.[1]

## Warum überhaupt ein Konjunkturprogramm?

Die staatliche Absicherung von krisenbedingten Risiken (Schutzschirm) ist oberstes Gebot in einer Wirtschafts-

1 Eine Bewertung des im Juni 2020 verabschiedeten Konjunkturpakets der Bundesregierung auf Grundlage der hier entwickelten Kriterien erfolgt aus Platzgründen an anderer Stelle (Krebs, 2020a).

krise und kann die ökonomischen Kosten solcher Krisen erheblich mindern. Private Haushalte und Unternehmen profitieren direkt von einer Politik der Risikoversicherung (Krebs, 2007) und indirekt durch eine Stabilisierung der gesamtwirtschaftlichen Nachfrage (Werning, 2015). Entsprechend hat die Bundesregierung im März und April 2020 schnell mit einem angemessenen Paket an Sofortmaßnahmen auf die wirtschaftspolitische Herausforderung reagiert: Kurzarbeit für Erwerbstätige als Schutz vor Arbeitslosigkeit, Zuschüsse für Solo-Selbstständige und kleinere Unternehmen als Kompensation für erlittene Umsatzverluste, vereinfachter Zugang zur Grundsicherung für Solo-Selbstständige, zinsgünstige KfW-Kredite für Unternehmen und staatliche Unternehmensbeteiligung zur Verhinderung einer Überschuldung.

Ein Schutzschirm für Wirtschaft und Gesellschaft ist der erste Schritt in einer umfassenden wirtschaftspolitischen Antwort auf die Corona-Krise. Ein solcher Schutzschirm federt Einkommensverluste ab und verhindert so einen dramatischen und irreversiblen Absturz der Wirtschaft. Doch er ist in der Regel nicht ausreichend, um Wirtschaft und Gesellschaft aus der Krise zu führen. Denn kein staatliches Stabilisierungsprogramm kann aufgrund möglicher Mitnahmeeffekte und negativer Anreizeffekte die privaten Einkommensverluste zu 100 % ersetzen. Es verbleibt also immer ein substanzieller Rückgang der gesamtwirtschaftlichen Nachfrage. Zudem sind private Haushalte und Unternehmen immer noch stark verunsichert und halten sich mit Konsum und Investitionen zurück. Schließlich werden einige gesundheitspolitische Restriktionen auf absehbare Zeit in Kraft bleiben, und viele Menschen haben ihr Konsumverhalten dauerhaft geändert.

Diese Überlegungen sprechen dafür, dass die deutsche Wirtschaft einen zusätzlichen Anschub benötigt, um zügig den Weg aus der Krise zu finden und einen Hysterese-Effekt zu vermeiden. Dieser wird in zahlreichen Studien belegt.[2] Für einen besonders starken Hysterese-Effekt in der Wirtschaftskrise 2020 spricht, dass die Corona-Pandemie das Verhalten privater Haushalte und Unternehmen unabhängig von den gesetzlichen Vorschriften und Geboten erheblich beeinflusst.

Die Bundesregierung hat also richtig gehandelt, wenn sie nach Verabschiedung der ersten Sofortmaßnahmen im März und April 2020 mit den Gesetzen „Begleitende Maßnahmen zur Umsetzung des Konjunktur-und Krisenbewältigungspakets“ (Bundesrat, 2020a) und „Feststellung eines Zweiten Nachtrags zum Bundeshaushaltsplan für das Haushaltsjahr 2020“ (Bundesrat, 2020b) Ende Juni 2020 ein Konjunkturpaket nachgeschoben hat. Doch welche Maßnahmen sollte ein Konjunkturpaket enthalten?

## Klassische Konjunkturpolitik

Die einfache keynesianische Antwort lautet, dass ein Konjunkturprogramm den Konsum über eine Steigerung der gesamtwirtschaftlichen Nachfrage ankurbeln sollte. Dabei liegt der Fokus häufig auf dem privaten Konsum. Wer mehr Geld in der Tasche hat, gibt auch mehr aus und hilft so mittelbar der ganzen Wirtschaft. So lautet verkürzt ein Kernargument für einfache Konjunkturhilfen. Zudem ist der private Konsum die größte Komponente der gesamtwirtschaftlichen Nachfrage, da gut die Hälfte aller Ausgaben für Güter und Dienstleistungen auf den privaten Konsum entfallen. Konsum ist also eine wichtige, aber nicht die einzige Nachfrage-Komponente. Öffentliche und private Investitionen steigern ebenfalls die gesamtwirtschaftliche Nachfrage und können so die Konjunktur ankurbeln. Deshalb betrachten viele Ökonomen ein öffentliches Investitionsprogramm als ein bewährtes Mittel zur Bekämpfung von Wirtschaftskrisen (Dullien et al., 2020; und Feld et al., 2020).

Ein gut strukturiertes Konjunkturprogramm sollte die zusätzlichen Staatsausgaben und Einnahmeausfälle durch eine Nettokreditaufnahme im öffentlichen Haushalt finanzieren. Ein Konjunkturprogramm muss also durch eine expansive Finanzpolitik begleitet werden, um die volle Wirkung entfalten zu können. Eine Finanzierung der Konjunkturmaßnahmen durch Steuererhöhungen oder Kürzungen an anderer Stelle wäre kontraproduktiv, denn es würde die positiven Konjunkturimpulse abschwächen oder sogar negieren. Dies zeigt, dass die Politik der schwäbischen Hausfrau in Rezessionen an ihre Grenzen stößt und in diesem Fall allen Generationen schaden kann.

Die Kreditfinanzierung eines Konjunkturprogramms widerspricht nicht dem Prinzip der Generationengerechtigkeit, wenn das Maßnahmenpaket krisenbedingten Schaden von Wirtschaft und Gesellschaft abwendet. Zudem kann ein kreditfinanziertes Konjunkturpaket die zukünftige Schuldenquote sogar drücken, wenn entweder der kurzfristige Wachstumsimpuls des Pakets sehr ausgeprägt ist oder die langfristigen Wachstumseffekte der im Paket enthaltenen Investitionsmaßnahmen stark sind. Die Krise ist also eine Situation, in der eine Neuverschuldung den Wohlstand und die fiskalischen Spielräume zukünftger Generationen verbessern kann.

2 Für eine Zusammenfassung der einschlägigen Literatur siehe Dullien et al. (2020).

### Moderne Konjunkturpolitik: Öffentliche Investitionen sind besser als ihr Ruf

In der aktuellen wirtschaftspolitischen Debatte zur Corona-Krise gibt es Stimmen, die den kurzfristigen Nutzen von Investitionsmaßnahmen – und insbesondere von öffentlichen Investitionsprogrammen – als eher gering einschätzen. Entsprechend haben Konsummaßnahmen wie z. B. die Mehrwertsteuersenkung oder der Kinderbonus ein relativ großes Gewicht im aktuellen Konjunkturpaket der Bundesregierung. Das Volumen der Investitionsmaßnahmen im Konjunkturpaket ist hingegen relativ moderat: Das geplante Gesamtvolumen von 130 Mrd. Euro für 2020/2021 enthält circa 80 Mrd. Euro für Konsummaßnahmen und 50 Mrd. Euro für Investitionsmaßnahmen nach haushaltsrechtlicher Abgrenzung. In Bezug auf das Investitionsvolumen fehlt dem Konjunkturpaket also ein bisschen der „Wumms“, auch wenn der Anstieg der Investitionsausgaben des Bundes 2020 von ursprünglich geplanten 42,9 Mrd. Euro auf jetzt geplanten 71,8 Mrd. Euro beachtlich ist.

Die häufig gehörte Kritik an Investitionsmaßnahmen als Konjunkturmaßnahmen, und der daraus abgeleitete Fokus auf den Konsum, ist aus ökonomischer Sicht überraschend. Denn es gibt eine Reihe von guten ökonomischen Gründen, warum Investitionsmaßnahmen in ihrer konjunkturellen Wirkung besser sind als ihr scheinbar schlechter Ruf in Teilen der Politik:

- Zum Ersten spricht die empirische Evidenz dafür, dass der konjunkturelle Impuls öffentlicher Investitionsausgaben in einer Krise sehr groß ist. Konkret findet eine Meta-Analyse zahlreicher empirischer Studien einen Fiskalmultiplikator öffentlicher Investitionen in Rezessionszeiten von knapp 2 (Gechert und Ranneberg, 2018). Zudem liegt dieser Wert erheblich über den Werten von Steuermultiplikatoren in Krisenzeiten.[3]

- Zum Zweiten ist Deutschland gut vorbereitet und muss kein neues Investitionsprogramm aufsetzen, sondern nur bestehende Programme und Pläne ausweiten. Beispielsweise hat der Bund in den letzten Jahren zahlreiche Programme zur Unterstützung der kommunalen Investitionen aufgelegt, die nur ausgeweitet bzw. verlängert werden müssen (Krebs, 2020b). Das bedeutet unter anderem, dass zusätzlich breitgestellte Finanzmittel zielgenau und zeitnah eingesetzt werden können. Das häufig gehörte Argument „mehr Geld bringt nichts“ trifft in diesem Fall nicht zu.

- Zum Dritten kann ein öffentliches Investitionsprogramm durch einen positiven Erwartungseffekt die Konjunktur ankurbeln, auch wenn die Umsetzung der in Auftrag gegebenen Investitionsprojekte einige Jahre dauern sollte. Ein solcher Erwartungseffekt öffentlicher Investitionsprogramme steht im Einklang mit dem empirischen Befund, dass öffentliche Investitionsausgaben einen sehr großen Konjunktureffekt ausweisen und die privaten Investitionen auch in der kurzen Frist steigern (Clemens et al., 2019). Zudem zeigen Analysen von Daten zur Zuversicht privater Unternehmen einen starken, positiven Erwartungseffekt öffentlicher Ausgabenprogramme – und insbesondere öffentlicher Investitionsprogramme – auf die gesamtwirtschaftliche Produktion in der kurzen Frist (Bachmann und Sims, 2012).

- Zum Vierten unterscheidet sich die Corona-Krise von vorherigen Krisen in fundamentaler Weise. Die wirtschaftspolitischen Maßnahmen des Konjunkturpakets werden zu einem Zeitpunkt greifen müssen, an dem gesundheitspolitische Restriktionen immer noch in Kraft sind. Es besteht somit die Gefahr, dass Maßnahmen zur Stimulierung der Konsumnachfrage die gesundheitspolitischen Maßnahmen konterkarieren. Zudem sind viele Menschen noch verunsichert und ihr Konsumverhalten hat sich geändert. Der Effekt von Maßnahmen zur Steigerung des privaten Konsums auf Beschäftigung und Wachstum ist daher in der aktuellen Krise geringer als in vorherigen Krisen, weil Arbeitsrestriktionen in Teilen des Konsumsektors und eine allgemeine Konsumzurückhaltung den keynesianischen Multiplikator-Effekt verkleinern können (Guerrieri et al., 2020).

### Moderne Konjunkturpolitik: Krise als Chance für den Transformationsschub

Öffentliche Investitionen sind nicht nur ein effektives Instrument zur Konjunkturstimulierung (Nachfrageseite), sondern sie stärken auch Produktionspotenziale und

3 Öffentliche Investitionen haben einen schwächeren Effekt auf die gesamtwirtschaftliche Aktivität, wenn durch einen Zinsanstieg private Investitionen verdrängt werden. Dieser Crowding-out-Effekt von defizit-finanzierten Staatsausgaben ist relativ groß in neu-keynesianischen Modellen, wenn die Zentralbank nicht entsprechend reagiert (Boehm, 2019). Das niedrige Zinsumfeld in Kombination mit der expansiven Geldpolitik der EZB legen nahe, dass in der aktuellen Situation dieser Crowding-out-Effekt klein sein sollte. Anders gesagt: Der positive Erwartungseffekt öffentlicher Investitionsausgaben dominiert den aktuell kleinen negativen Zinseffekt, und öffentliche Investitionen steigern daher private Investitionen in der kurzen Frist. Diese These wird von der empirischen Evidenz gestützt (Clemens et al., 2019). In der langen Frist führen dauerhafte Steigerungen der öffentlichen Investitionsausgaben zu einem erheblichen Anstieg der gesamtwirtschaftlichen Produktion, weil der öffentliche Kapitalstock steigt und öffentliche und private Investitionen komplementäre Produktionsfaktoren sind (Krebs und Scheffel, 2017).

können nachhaltiges, inklusives Wachstum schaffen (Angebotsseite). Die folgenden zwei angebotsseitigen Argumente sprechen für eine Wirtschaftspolitik, die die Krise als Chance nutzt, um die öffentlichen Investitionen dauerhaft zu steigern:

- Erstens stand bereits vor der Krise fest, dass Deutschland ökologisch nachhaltiger wirtschaften muss, um die Klimaziele zu erreichen. Die Fridays-for-Future-Demonstrationen und das im Herbst 2019 verabschiedete Klimaprogramm 2030 sind eindeutige Anzeichen, dass diese Erkenntnis bei der Mehrheitsgesellschaft angekommen ist und die Politik versucht, angemessen zu reagieren. Zudem haben viele Menschen das Gefühl, dass die Lebenschancen nicht gerecht verteilt sind. Dies ist nicht nur ein Gefühl, sondern in gewissen Bereichen der Gesellschaft auch die Realität.[4] Anders gesagt: Deutschland braucht eine sozial-ökologische Transformation von Wirtschaft und Gesellschaft, um die Generationengerechtigkeit zu gewährleisten und die soziale Gerechtigkeit zu stärken. Eine gut ausgebaute öffentliche Infrastruktur ist Voraussetzung für eine solche sozial-ökologische Transformation.

- Zweitens bietet die Corona-Krise eine Chance, durch ein großangelegtes Investitionsprogramm den notwendigen Transformationssprung auf einen klimafreundlichen und sozial ausgewogenen Wachstumspfad einzuleiten. Denn in einer Krise sind die Opportunitätskosten der Zukunftsinvestitionen geringer als in normalen Zeiten. Zum Beispiel werden in den kommenden Monaten die Produktionskapazitäten in vielen Betrieben nicht voll ausgelastet sein und die frei gewordene Zeit kann genutzt werden, um alte Anlagen durch klimafreundliche Alternativen zu ersetzen und notwendige Umschulungen des Betriebspersonals durchzuführen. Zudem werden viele der Schulabgänger 2020 voraussichtlich keinen Ausbildungsplatz finden, weil die Bewerbungsphase in eine Zeit fällt, in der sich die Unternehmen mit Neueinstellungen zurückhalten. Aus ökonomischer Sicht ist es sinnvoll, in solchen Zeiten staatliche Sonderprogramme mit verbesserten Konditionen zur Ausbildung von Fachkräften in Berufen wie frühe Bildung und Pflege aufzulegen.

4 Beispielsweise beträgt in Deutschland das erwartete Lebenseinkommen von Kindern aus weniger privilegierten Familien nur die Hälfte des Lebenseinkommens von Akademiker-Kindern (Krebs und Scheffel, 2017). Zudem beträgt das erwartete Lebenseinkommen von Frauen mit Kindern weniger als die Hälfte des Lebenseinkommens von Männern mit Kindern (Bönke et al., 2020).

Prof. Tom Krebs, Ph.D., ist Professor für Makroökonomik und Wirtschaftspolitik an der Universität Mannheim.


Literatur

Bachmann R Sims E Confidence and the transmission of government spending shocks Journal of Monetary Economics 2012 59 3 235 249 10.1016/j.jmoneco.2012.02.005
Boehm C Government Consumption and Investment: Does the Composition of Purchases Affect the Multiplier? Journal of Monetary Economics 2019 53 7 1341 1359
Bönke, T., R. Glaubitz, K. Göbler, A. Harnack, A. Pape und M. Wetter (2020), Wer gewinnt, verliert?, Studie im Auftrag der Bertelsmann Stiftung.
Bundesrat (2020a), Begleitende Maßnahmen zur Umsetzung des Konjunktur- und Krisenbewältigungspakets, Drucksache 389/20 (Beschluss vom 3.3.2020), https://www.bundesrat.de/SharedDocs/drucksachen/2020/0301-0400/389-20(B).pdf?__blob=publicationFile&v=1 (6. Juli 2020).
Bundesrat (2020b), Feststellung eines Zweiten Nachtrags zum Bundeshaushaltsplan für das Haushaltsjahr 2020 Drucksache 388/20 (Beschluss vom 3.7.2020), https://www.bundesrat.de/SharedDocs/drucksachen/2020/0301-0400/388-20(B).pdf?__blob=publicationFile&v=1 (6. Juli 2020).
Clemens M Goerge M Michelsen C Public investment a key prerequisite for private sector activity DIW Weekly Report 2019 31 255 261
Dullien, S. und S. Gechert (2020), Stellungnahme für die Anhörung im Finanzausschuss des Deutschen Bundestages am 22.6.2020.
Dullien, S., M. Hüther, T. Krebs, B. Praetorius und K. Spieß (2020), Weiter Denken: Ein Nachhaltiges Investitionsprogramm als tragende Säule einer gesamtwirtschaftlichen Stabilisierungspolitik, IMK Policy Brief, 90.
Feld, L., V. Grimm, M. Schnitzer, A. Truger, V. Wieland (2020), So kann sich die Wirtschaft erholen, Süddeutsche Zeitung, 22. Mai.
Gechert S Rannenberg A Which Fiskal Multipliers are Regime-Dependent? A Meta-Regression Analysis Journal of Economic Surveys 2018 32 4 1160 1182 10.1111/joes.12241
Guerrieri, V., G. Lorenzoni, L. Straub und I. Werning (2020), Macroeconomic Implications of COVID-19: Can Negative Supply Shocks Cause Demand Shortages?, NBER Working Paper, Nr. 26918.
Krebs T Job Displacement Risk and the Cost of Business Cycles American Economic Review 2007 97 3 664 686 10.1257/aer.97.3.664
Krebs, T. (2020a), Schriftliche Stellungnahme für die Anhörung im Haushaltsausschuss des Deutschen Bundestages zum Entwurf eines Gesetzes über die Feststellung eines Zweiten Nachtragshaushalts für das Haushaltsjahr 2020 und zum Entwurf eines Gesetzes über begleitende Maßnahmen zur Umsetzung des Konjunktur- und Krisenbewältigungspakets.
Krebs, T. (2020b), Schriftliche Stellungnahme für die Anhörung im Haushaltsausschuss des Deutschen Bundestages zu den Anträgen der Fraktionen DIE LINKE, FDP und BÜNDNIS90/DIE GRÜNEN zum Thema Schuldenbremse und Investitionen.
Krebs, T. und M. Scheffel (2017), Öffentliche Investitionen und inklusives Wachstum in Deutschland, Studie im Auftrag der Bertelsmann Stiftung.
Werning, I. (2015), Incomplete Markets and Aggregate Demand, Working Paper, 21448.
